# Bowman–Birk Inhibitor Mutants of Soybean Generated by CRISPR-Cas9 Reveal Drastic Reductions in Trypsin and Chymotrypsin Inhibitor Activities

**DOI:** 10.3390/ijms25115578

**Published:** 2024-05-21

**Authors:** Won-Seok Kim, Jason D. Gillman, Sunhyung Kim, Junqi Liu, Madhusudhana R. Janga, Robert M. Stupar, Hari B. Krishnan

**Affiliations:** 1 Division of Plant Science and Technology, University of Missouri, Columbia, MO 65211, USA; wonseokk@missouri.edu (W.-S.K.); jeongs@missouri.edu (S.K.); 2 Plant Genetics Research Unit, US Department of Agriculture-Agricultural Research Service, Columbia, MO 65211, USA; jason.gillman@usda.gov; 3 Department of Agronomy and Plant Genetics, University of Minnesota, St. Paul, MN 55108, USA; liuxx162@umn.edu (J.L.); stup0004@umn.edu (R.M.S.); 4 Institute of Genomics for Crop Abiotic Stress Tolerance, Texas Tech University, Lubbock, TX 79409, USA; mjanga@ttu.edu

**Keywords:** soybean, *Glycine max* (L. (Merr)), antinutritional factors, Bowman–Birk inhibitor, CRISPR-Cas9 enabled mutagenesis

## Abstract

Despite the high quality of soybean protein, raw soybeans and soybean meal cannot be directly included in animal feed mixtures due to the presence of Kunitz (KTi) and Bowman–Birk protease inhibitors (BBis), which reduces animal productivity. Heat treatment can substantially inactivate trypsin and chymotrypsin inhibitors (BBis), but such treatment is energy-intensive, adds expense, and negatively impacts the quality of seed proteins. As an alternative approach, we have employed CRISPR/Cas9 gene editing to create mutations in *BBi* genes to drastically lower the protease inhibitor content in soybean seed. Agrobacterium-mediated transformation was used to generate several stable transgenic soybean events. These independent CRISPR/Cas9 events were examined in comparison to wild-type plants using Sanger sequencing, proteomic analysis, trypsin/chymotrypsin inhibitor activity assays, and qRT-PCR. Collectively, our results demonstrate the creation of an allelic series of loss-of-function mutations affecting the major *BBi* gene in soybean. Mutations in two of the highly expressed seed-specific *BBi* genes lead to substantial reductions in both trypsin and chymotrypsin inhibitor activities.

## 1. Introduction

Soybean *Glycine max* (L.) Merr) is grown worldwide as a rotation partner for grain crops, particularly maize, due to an ability to fix atmospheric nitrogen, high seed yield potential, relatively few major biotic pathogens, and relatively low input costs [1,2]. Soybean seeds contain far more protein than other legumes (on average 40%), and soybean meal is often the first choice for swine and poultry feed mixtures due to a balanced (albeit suboptimal) amino acid profile. Soybean meal, produced after extraction of seed oil, has a relatively low cost and is of high quality compared to other competing protein meal sources [3].

Despite the high quality of soybean protein, raw soybeans and soybean meal cannot be directly included in animal feed mixtures due to anti-nutritional compounds that interfere with digestion, cause pancreatic inflammation, and effectively reduce animal weight gain. Soybean seeds contain two major classes of proteinaceous anti-nutritional factors [4], which were named after the scientists that discovered them: (1) Kunitz trypsin inhibitors [5] (KTis), which inactivate the digestive enzyme trypsin, and (2) Bowman–Birk inhibitors [6] (BBis), which inactivate both trypsin and chymotrypsin animal digestive enzymes [7]. In soybean, three KTi isoforms (KTi1, KTi2, and KTi3) have been reported [8,9,10]. There are at least 11 different *BBi*-encoding genes present in the soybean genome; however, expression analysis of RNAseq data reveals that only three of them are expressed at appreciable levels in developing seeds (Figure S1).

Experiments have conclusively shown that ingestion of high levels of active protease inhibitors is responsible for reduced animal weight gain and can even result in hypoglycemia, pancreatic hypertrophy, or liver damage [11]. To avoid these issues and permit the inclusion of soybean meal at high levels in animal feed, KTi and BBi anti-nutritional proteins in typical soybean seeds must be inactivated by heat treatment, which has been shown to reduce, but not eliminate, ANF activity [7]. Heat inactivation allows soybean meal to be included at moderately high levels in chicken and swine feed mixtures. While this step meets the needs of the industry, such treatment is energy-intensive, adds expense, and requires a balance between sufficiently reducing ANF activity and negatively impacting the quality of seed proteins [3,11].

One success story in genetically reducing soybean’s trypsin inhibitor activity was the identification and analysis of a soybean accession (PI 157740), which features a marked reduction (~40%) in trypsin inhibitor activity. This genetic source has been examined through both genetic analysis and animal feeding trials. Raw extruded protein meal lacking KTi3 protein was found to result in superior animal weight gain compared with raw soybeans [12] but not to a degree that could eliminate the need for heat inactivation. In our previous research, a soybean line (PI 68679) with reductions in trypsin inhibition due to a mutation affecting the KTi1 gene was identified [13]. Using hybridization, the *KTi-1* mutation (PI 68679) was combined with the previously identified *KTi-3* mutation (PI 542044), to yield soybean lines that lack both KTi1 and KTi3 protein. These soybean lines have lower trypsin inhibitor activity than the currently available *KTi-3* mutant [13]. Unfortunately, all lines that have reduced KTi levels unexpectedly had unacceptable levels of trypsin inhibitor activity due to overaccumulation of BBi proteins in seeds of all mutant lines (*KTi1*, *KTi3*, and *KTi1*/*KTi3*).

To develop soybean lines that require extremely low or no heat-inactivation processing, we hypothesize that both *KTi* and *BBi* gene expression in seeds must be reduced or ideally eliminated. Naturally occurring *BBi* mutants have been identified in perennial soybean collections [14]. However, to the best of our knowledge, no naturally occurring *G. max* mutant or germplasm entry has been reported with substantively reduced BBi accumulation or activity. However, one report indicated that transgenic expression of a modified protein can lower BBi activity and resulted in viable plants that were able to produce seeds [15].

CRISPR/Cas9 gene editing [16,17] is now a routine practice for inducing targeted loss-of-function mutations in soybean [17,18,19]. This method could be leveraged to induce mutations of soybean seed *BBi* genes and decrease or eliminate their accumulation in seeds. In this study, we report on the creation of an allelic series of loss-of-function mutations affecting the major *BBi* seed-expressed genes in soybean, and mutation of two genes was shown to lead to substantial reductions in both trypsin and chymotrypsin inhibitor activities due to loss-of-function mutations affecting BBi genes.

## 2. Results

### 2.1. Generation of BBi Mutants by CRISPR/Cas9 Gene Editing

**Figure 1:**
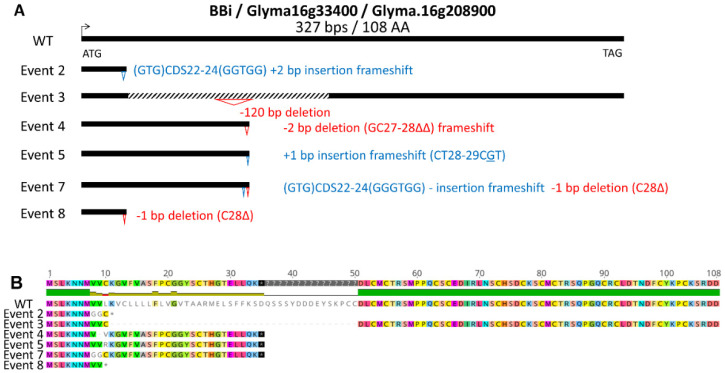
CRISPR-Cas9 mutations identified in Glyma.16g208900, the most highly expressed BBi isoform. (**A**) Summary of mutations identified in six independent events. (**B**) Effect of CRISPR—CAS9-induced deletions, insertions, and frameshift mutations on the amino acid sequence. * = stop codon.

**Figure 2:**
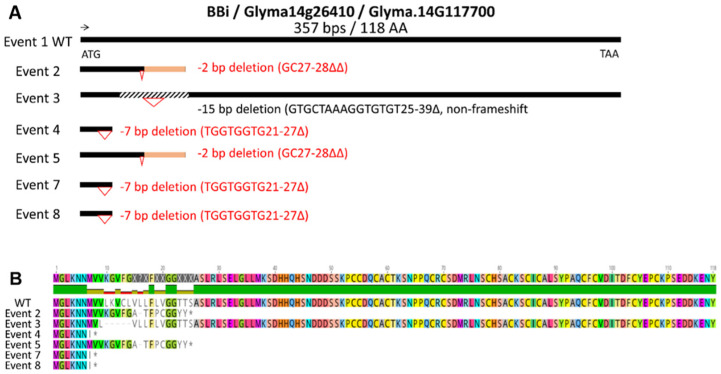
CRISPR-Cas9 mutations identified in Glyma.14G117700, the second-most highly expressed BBi isoform. (**A**) Summary of mutations identified in six independent events. (**B**) Effect of CRISPR—CAS9-induced deletions, insertions, and frameshift mutations on the amino acid sequence. * = stop codon.

At least 11 *BBi*-encoding genes exist in the soybean genome; however, transcriptome analysis implicates only one major and two minor seed-specific *BBi* genes in developing soybean seeds (Supplementary Figure S1) [20]. Because *BBi* genes share extensive sequence homology, we designed target sites that enabled simultaneous knockout of all three seed-expressed genes, and two CRISPR/Cas9 expression cassettes were designed, which differed in use of either single or double guideRNA (Supplementary Figure S2). Utilizing these two CRISPR/Cas9 expression cassettes, we generated several independent transgenic events by *Agrobacterium*-mediated soybean transformation (Figure 1A). Six transgenic events were grown in the greenhouse, and leaf tissue from these plants was utilized for the isolation of genomic DNA. Sanger sequencing of PCR products generated by utilizing primers that are specific for the most abundantly expressed *BBi* gene (Glyma.16g208900) identified several mutations in this gene. Figure 1A shows a schematic summary of mutations identified in these six independent events leading to deletions, insertions, and frameshift mutations on the amino acid sequence (Figure 1B and Figure S3). Similarly, we also sequenced PCR products generated by utilizing primers that are specific for the second-most abundantly expressed *BBi* gene (Glyma.14G117700). This analysis revealed several mutations that introduced deletions, insertions, and frameshift mutations on the amino acid sequence (Figure 2A,B and Figure S3).

### 2.2. SDS-PAGE Analysis of Seed Proteins of CRISPR/Cas9 Transgenic Plants

**Figure 3:**
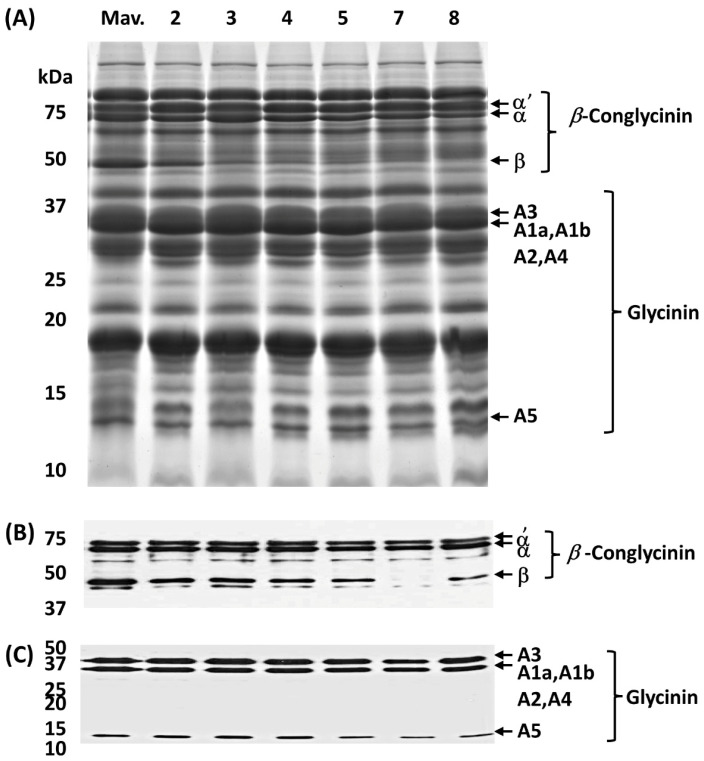
Proteomic analysis of total seed proteins from CRISPR-Cas9 BBi mutant and control lines. (**A**) Sodium dodecyl sulfate polyacrylamide gel electrophoresis of total seed proteins visualized with Coomassie Blue; (**B**) immunoblot using *β*-conglycinin-specific antibodies; (**C**) immunoblot using glycinin-specific antibodies. Samples in order are ‘Maverick’ (Mav., non-transgenic control); 2 = Event 2; 3 = Event 3; 4 = Event 4; 5 = Event 5; 7 = Event 7; 8 = Event 8.

First, we wanted to compare the total seed protein profile between the wild-type and CRISPR/Cas9 transgenic plants. For this purpose, we first isolated total seed proteins from the wild-type and the six independent transgenic events and resolved them by SDS-PAGE (Figure 3A). A visual examination of the total seed protein profile between wild-type and transgenic events did not reveal any drastic alterations between them (Figure 3A). All soybean seeds examined accumulated the two most abundant seed storage proteins (7S β-conglycinin and 11S glycinin). However, the accumulation of the β-subunit of β-conglycinin, a 52 kDa glycoprotein, in all transgenic events was noticeably lower when compared to the wild type (Figure 3A). To substantiate these changes, we performed immunoblot analysis using antibodies raised against the 7S β-conglycinin and 11S glycinin antibodies. The 7S β-conglycinin antibody reacted against all three subunits. The accumulation pattern of the α′ and α subunits of β-conglycinin was similar between the wild-type and the six CRISPR/Cas9 transgenic events (Figure 3B). In contrast, the accumulation of the β-subunit of β-conglycinin was much lower in all transgenic events when compared to the wild type, and for one transgenic event (Figure 3B, lane 7) only an extremely faint band was detected. We also performed immunoblot analysis to quantify any changes in the 11S glycinin accumulation between wild-type and CRISPR/Cas9 transgenic events. The glycinin antibody recognized three glycinin species (A3; A1a, A1b, A2, and A4; A5). In contrast to the β-subunit of β-conglycinin, the accumulation of 11S glycinin showed little variation in abundance between the wild-type and the CRISPR/Cas9 transgenic events (Figure 3C).

### 2.3. Immunoblot Analysis Reveals the Absence of BBi in the Seeds of Transgenic BBi Mutants

**Figure 4:**
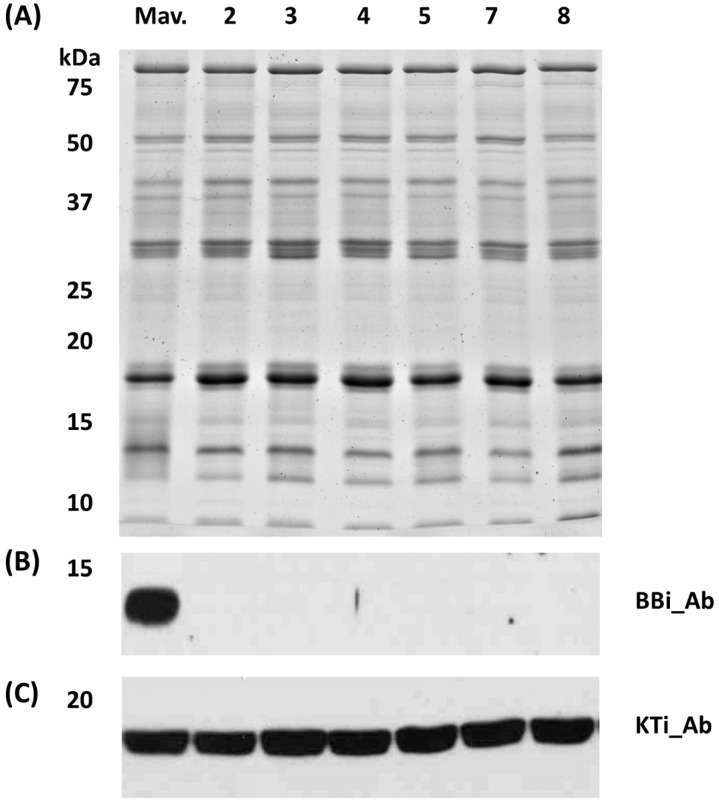
Proteomic analysis of soluble seed proteins from CRISPR-Cas9 BBi mutant and control lines. (**A**) Sodium dodecyl sulfate polyacrylamide gel electrophoresis of calcium fractionated soluble seed proteins visualized with Coomassie Blue; (**B**) immunoblot using Bowman–Birk inhibitor (BBi)-specific antibodies; (**C**) immunoblot using KTi-specific antibodies (KTis). Samples in order are ‘Maverick’ (Mav., non-transgenic control); 2 = Event 2; 3 = Event 3; 4 = Event 4; 5 = Event 5; 7 = Event 7; 8 = Event 8.

To examine the accumulation of BBi in seeds of the CRISPR/Cas9 transgenic events, we performed Western blot analysis utilizing BBi-specific peptide antibodies [21]. A protein fraction enriched for protease inhibitors obtained by the calcium fractionation method [22] was utilized for the immunoblot analysis and transferred to a nitrocellulose membrane. The nitrocellulose membrane when challenged with soybean BBi-specific antibodies failed to detect the accumulation of this protein in all transgenic events. In contrast, the BBi antibody reacted against a 11–12 kDa protein in the wild-type plant (Figure 4B). We also examined the accumulation of KTi, which also contributes significantly to the overall protease inhibitor of soybean seed, by immunoblot analysis, via antibodies raised against soybean KTi [9]. Immunoblot analysis clearly demonstrated normal accumulation of KTi proteins in all the plants examined in our study (Figure 4C).

### 2.4. Chymotrypsin and Trypsin Inhibitor Activities Are Drastically Reduced in CRISPR/Cas9 Gene-Edited Transgenic BBi Mutants

Since our immunoblot analysis clearly showed the absence of BBi protein accumulation in the CRISPR/Cas9 gene-edited transgenic *BBi* mutants, we also wanted to examine protease inhibitor activities in these plants. The chymotrypsin inhibitor activity (CIU) was measured using N-Succinyl-Ala-Ala-Pro-Phe p-nitroanilide as a substrate and is expressed as CIU /mg seed powder. The wild-type soybean seed contained 17.2 CIU, while in transgenic seeds the activity ranged from 2.89 to 15.9 CIU. Out of the six transgenic events examined in this study, four had drastic reductions; we saw a 2.8–6.0-fold reduction in CIU compared to the wild type (Table 1). Since soybean BBi proteins can inhibit both chymotrypsin and trypsin, we also measured the trypsin inhibitor activity (TIU) using the same seed extracts (Table 1), with Nα-Benzoyl-DL-arginine 4-nitroanilide hydrochloride as a substrate. The trypsin inhibitor activity in the wild-type soybean seeds averaged 57.25 TIU/mg seed powder, whereas we saw reduced activities in mutant lines ranging from a low of 14.46 (Event 8) to a high of 35.7 TIU (Event 5) for different transgenic *BBi* events. This corresponds to a 1.6–4.0-fold reduction in TIU activities. TIU activity in all examined transgenic *BBi* mutants was significantly lower than in wild-type seeds. Moreover, in four of the transgenic events (Events 2, 4, 7, and 8; Table 1) we observed drastic reductions in the TIU activities ranging from 25.3 to 41.9% of the TIU activities found in wild-type seeds. Interestingly, the reduction in TIU activity in these *BBi* mutants is more significant than we observed for a naturally occurring soybean frameshift *KTi-3* mutant (Table 1). CIU and TIU activities in seeds of the mutant and the control line were fully concordant when activities were expressed as TIU/mg seed powder or as TIU/mg seed protein (Table 1).

### 2.5. qRT-PCR Analysis Confirms the Absence of Expression of the Abundant BBi Genes in CRISPR/Cas9 Gene-Edited Transgenic BBi Mutants

**Figure 5:**
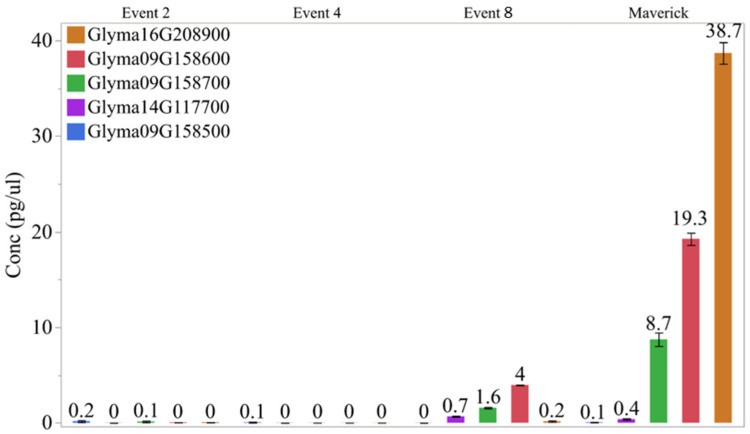
RT-PCR analysis of T1 seeds with CRISPR-Cas9 BBi mutant and control lines.

We also examined the expression of the two most abundantly expressed *BBi* genes (Glyma.16G208900 and Glyma.14G117700) along with a few other *BBi* genes (Glyma.09G158500, Glyma.09G158600, and Glyma.09G158700) in the CRISPR/Cas9 gene-edited transgenic *BBi* mutants. Total RNA was isolated from mid-stage (35 days after flowering) developing T1 soybean seeds and used for qRT-PCR analysis. The gene-specific primers used in our study are shown in Table S1. In the wild-type soybean seeds, we could detect the expression of most of these *BBi* genes. At this stage of seed development, we observed high level expression of Glyma.16G208900, Glyma.14G117700, and Glyma.09G158700 (Figure 5). In contrast, the expression of these abundantly expressed *BBi* genes was essentially not detected in two of the transgenic events (events 2 and 4), while several-fold lower expression was detected in one of the mutants (event 8).

## 3. Discussion

In this study, we generated several stable transgenic soybean lines that exhibit the lowest levels of trypsin and chymotrypsin inhibitor activities that have been reported in the literature. Several of our CRISPR-Cas9 *BBi* lines have much lower trypsin inhibitor activity (62% to 75% lower) when compared to control plants. The relative abundance of KTi proteins in soybean seeds is higher than that of BBis [23]. It has been reported that BBi proteins are more heat stable than KTi proteins since the BBi contains multiple cysteine residues leading to conformational change in the BBi during heating [24]. Since our CRISPR-Cas9 *BBi* lines contain undetectable levels of BBi proteins, as evidenced by immunoblot analysis, one would expect that these newly developed *BBi* mutant lines may require only minimal heat treatment.

Until now, no soybean null mutants with reduced or ablated BBi proteins have been identified in *G. max* germplasm. However, *BBi* mutants have been identified in several perennial *Glycine* spp. [14]. Unfortunately, substantial hybridization barriers exist between perennial *Glycine* spp. and *G. max*, which has hampered the development of *G. max BBi* mutants by traditional breeding. Attempts have been made to lower the protease inhibitor activity in soybean seeds through overexpression of a mutated *BBi* gene [15]. The transgenic seeds generated in that study revealed a 20–50% reduction in protease inhibitory activity. In contrast, we have developed several CRISPR-Cas9-mutated *BBi* lines that showed remarkable reduction (ranging from 63 to 84%) in chymotrypsin inhibitor activity when compared to the wild-type seeds. Even though our CRISPR-Cas9 constructs were targeted to knockout seed-specific *BBi* genes, their pronounced effect on lowering the trypsin inhibitor is striking. This drastic reduction in trypsin inhibitor activity is not mediated by a reduction in the accumulation of KTi proteins in the CRISPR-Cas9 *BBi* lines. Our immunoblot analysis (Figure 4) clearly demonstrates that KTi proteins accumulate to the similar levels in *BBi* mutant lines as in wild-type seeds. Thus, despite the presence of functional versions of all three KTi seed-expressed genes, we saw a significant reduction in the trypsin inhibitor activity. This reduction is solely because of *BBi* mutations and unexpectedly revealed that *BBi* contributes significantly to overall trypsin inhibitor activity in soybean seeds.

Our immunoblot analysis (Figure 4) and qRT-PCR analysis (Figure 5) confirmed that the gRNA we employed in our study to knockout the seed-specific *BBi* genes was very effective. In addition to eliminating the expression of the two most abundant *BBi* genes (Glyma.16G208900 and Glyma.14G117700), other *BBi* genes (Glyma.09G158500, Glyma.09G158600, and Glyma.09G158700) were also eliminated in some the CRISPR-Cas9 *BBi*-induced mutant lines. This was an expected result as *BBi* genes share extensive sequence homology, and constructs were designed to target multiple *BBi* genes. The full number of mutated genes is unknown and will likely require creation and analysis of segregated populations to discern the number of mutated genes and their relative contributions to trypsin and chymotrypsin inhibition activities. One intriguing prospect is the combination of null mutations for both seed-expressed KTi genes and our newly identified null mutations affecting all seed-expressed *BBi* genes.

Our study also reveals proteome alteration due to knockout of *BBi* genes. One-dimensional gel analysis of total seed proteins from *BBi* mutant lines reveals lower accumulation of the β-subunit of β-conglycinin. The accumulation of this protein is influenced by the nitrogen and sulfur status of the plant [25,26]. The accumulation of the β subunit of β-conglycinin, a protein that is deficient in sulfur containing amino acids, is elevated when the plants are grown under limited sulfur supply conditions [27,28,29]. In the current study, there is a drastic reduction in the accumulation of BBis in all CRISPR-Cas9-mutated *BBi* lines. BBi contains 14 cysteine residues and acts as a main sink for sulfur storage in the seed [30,31]. In the absence of BBi accumulation, cysteine that would be normally incorporated in BBis will be freely available. We speculate that the reduction in the accumulation of the β-subunit of β-conglycinin could be related to an increase in the availability of cysteine in the seeds, which would be incorporated into other, normally lower abundance seed-expressed proteins. Our suggestion for this possibility is strengthened by previous reports that has shown that an increased availability of sulfur amino acids in transgenic soybean plants reduces the accumulation of β subunit of β-conglycinin [32]. However, this hypothesis remains to be rigorously tested through greenhouse and/or field studies. Although we clearly demonstrate the creation of an allelic series of *BBi* null mutations through CRISPR Cas9 mutagenesis, we acknowledge that we do not currently know what impact, if any, such mutations (or a combination of mutations) will have on field agronomic performance or seed yield. Similar work with *KTi-3* mutant lines did not identify any deleterious impacts on whole-plant agronomic traits or seed yield [33].

Our results open the possibility that breeding lines could be developed that require substantially less heat treatment, which would save both money and energy used to heat treat soybeans. One study performed with KTi-3 and lectin-free seeds demonstrated that partial loss of trypsin/chymotrypsin inhibitors resulted in superior animal weight gain performance as compared to conventional soybean meal; however, both were inferior to heat-processed soybean meal [11]. Given our finding that our *BBi* mutants had reduced trypsin inhibitor activity equivalent to the *KTi-3* mutation (Table 1), the *BBi* allelic series we have created may open the door to even greater reductions in trypsin and chymotrypsin inhibitor content in animal feed mixtures and for human food products, such as tofu and soymilk, while also reducing the energy required for heat processing.

## 4. Materials and Methods

### 4.1. Chemicals

Most chemicals and reagents used in this study were of analytical grade. Chemicals for electrophoresis, including acrylamide, bis acrylamide, SDS, TEMED, and ammonium persulfate, were purchased from GE healthcare (Piscataway, NJ, USA). Tris-HCL, trypsin, chymotrypsin, β-mercaptoethanol, Nα-Benzoyl-DL-arginine p-nitroanilide hydrochloride, and N-Succinyl-Ala-Ala-Pro-Phe p-nitroanilide were purchased from Sigma-Aldrich (St. Louis, MO, USA).

### 4.2. Analysis of Expression of BBi Genes Using Public Data Repositories

Soybase.org is a repository for data from multiple experimental datasets for soybean. We identified protein coding genes in the ‘Williams 82’ genome soybean with the annotation PFAM PF00228 (Bowman–Birk serine protease inhibitor family) as targets. Previously collected data from the ‘RNA-Seq Atlas’ [20] was used to prioritize target *BBi* genes for CRISPR-Cas9 constructs, gene sequencing, and RT-PCR analysis. In developing seeds, two genes, Glyma.14g117700 and Glyma.16g208900, represent between 17.0 and 31.3% and 68.5 and 82.8% of the total detectable *BBi* gene expression from seeds samples between 21 and 42 days after flowering.

### 4.3. Generation of CRISPR/CAS9-Induced BBi Knockout Soybean Mutants

For CRISPR/Cas9-mediated mutagenesis of the BBi genes in soybean, two single gRNAs were designed to target the two major expressed BBi genes and three other *BBi* genes. gRNA1: (5′-GAACAACATGGTGGTGCTAAAGG-3′) was a common target in five *BBi* genes (Glyma.09G158500; Glyma.14G117700; Glyma.09G158600; Glyma.09G158700; Glyma.16G202800), and gRNA2 (5′-CACATGCAGAGATCACAGCATGG-3′, reverse complimentary of the sense strand sequence 5′-CCATGCTGTGATCTCTGCATGTG-3′) was a common target in only 3 *BBi* genes (Glyma.09G158600; Glyma.09G158700; Glyma.16G202800). Two plasmid vectors were constructed: the first plasmid contained only gRNA1, and the second plasmid contained both gRNA1 and gRNA2. For cloning of the *BBi* gRNAs in the T-DNA vector, a two-step Golden Gate Assembly was performed firstly in the module vector pMOD_B2103 and then subsequently in the destination T-DNA vector pTRANS230d as described previously [18,34]. Recombinant pMOD_B2103_BBi gRNAs plasmids harboring *BBi* gRNAs were verified by Sanger sequencing of the gRNA cassette with primers flanking the gRNAs: the forward primer TC320 (5′-CTAGAAGTAGTCAAGGCGGC-3′) and reverse primer TC089R (5′-GGAACCCTAATTCCCTTATCTGG-3′), respectively. The second Gloden Gate Assembly reaction was composed of the recombinant pMOD_B2103_BBi gRNAs, pMOD_A0521, pMOD_C2906, and a destination T-DNA vector pTRANS230d. Final CRISPR/Cas9_BBi gRNA constructs in the destination T-DNA vector were designated as pTRANS230/BBi_1gRNA and pTRANS230/BBi_2gRNA, respectively. The CRISPR constructs were mobilized into a disarmed *Agrobacterium tumefaciens* strain EHA105 by a heat shock procedure.

### 4.4. Soybean Transformation

The soybean genotype ‘Maverick’ [35] was transformed using *Agrobacterium*-mediated transformation of half-seed explants. The transformation protocol was modified from Zeng et al. [36]. The process began by germinating sterilized seeds for 16 h in Petri dishes on germination media. Then, half seed explants were made from the seeds and treated with an *Agrobacterium* culture [37]. After 5 days of co-cultivation, the explants were transferred to shoot induction medium. Subsequently, the explants underwent two rounds of subcultures before being moved into shoot elongation medium. Elongated shoots were then cut from the explants and used for rooting. After 2 weeks, the rooted plants were transferred into soil. To identify putative transgenic soybean plants, a leaf paginating assay was performed with 100 mg L^−1^ glufosinate–ammonium solution on leaves at three stages. Herbicide-resistant plants were cultivated in greenhouses under controlled conditions, with a 16 h day at 28 °C and 8 h night at 24 °C until T1 seeds were harvested.

### 4.5. Protein Extraction and 1-D Electrophoresis

Total soybean seed protein extraction and subsequent separation of proteins by 1-D SDS-PAGE were carried out as previously described [38]. Briefly, dry soybean seeds were ground to a fine power with a mortar and pestle and extracted with 1 mL of sodium dodecyl sulfate (SDS) sample buffer (60 mM Tris-HCl, pH 6.8, 2% SDS, 10% glycerol and 5% 2-mercaptoethanol). Tubes were vortexed (Vortex Genie, Scientific Industries, San Diego, CA, USA) for 10 min at room temperature and then heated to 100 °C for 5 min followed by centrifugation (Eppendorf, Beckman Coulter, Inc., Indianapolis, IN, USA) at 15,800× *g* for 5 min. The supernatant is the total seed protein fraction. This fraction was resolved by 13.5% SDS-PAGE gels using a Hoefer SE 260 minigel apparatus (Amersham Biosciences, Piscataway, NJ, USA). Electrophoretic separation of proteins was achieved with 20 mA per gel (constant current). SDS-PAGE-separated proteins were visualized using Coomassie Blue R-250 solution (0.3% Coomassie Brilliant Blue R-250, 45% methanol, and 10% glacial acetic acid).

### 4.6. Real-Time qRT-PCR Analysis of BBi Genes in Developing Soybean Seeds

Total RNA from 35 days after anthesis soybean seeds was extracted using TRIzol reagent (Invitrogen, Waltham, MA, USA) according to the manufacturer’s instructions. Real-time qRT-PCR was conducted using a LightCycler 480 II instrument (Roche, Indianapolis, IN, USA). Each qRT-PCR reaction was conducted with two replications in 20 µL using the QuantiNova SYBR Green RT-PCR Kit (Qiagen, Hilden, Germany) following the manufacturer’s instructions. The cycling condition of absolute quantification analysis were 10 min at 50 °C then 2 min 95 °C for cDNA synthesize followed by 40 cycles of 20 s at 95 °C and 40 s at 60 °C, with the data acquired at 60 °C in the green channel. The reference gene (F-box protein, Glyma.12G051100) was amplified by RT-PCR from Maverick soybean seed total RNA with a gene-specific primer set (Supplementary Table S1). The amplified reference gene RT-PCR fragment was purified from agarose gel, and the standard was calculated based on the purified fragment concentration and molecular weight. The reference gene was subjected to a 10-fold serial dilution (1.00 × 10^1^ to 1.00 × 10^−10^) with DEPC. For absolute quantification analysis, five standard dilutions from 1.00 × 10^−5^ to 1.00 × 10^−9^ for a standard curve were used for quantifying both target and reference gene expression in cDNA samples.

### 4.7. Screening of Putative Transgenic Plants and Identification of CRISPR-CAS9-Induced Mutations

Twenty seeds from 10 individual T0 *BBi* events were planted in the Sears Plant greenhouse in 2-gallon pots containing Pro-Mix^®^ HP Mycorrhizae™ (Premier Tech, Quakertown, PA, USA). The plants were fertilized with Osmocote Plus (Scotts, Marysville, OH, USA) according to the manufacturer’s recommendations. Greenhouse settings were 16 h daylength and 30 °C/18 °C day/night temperatures. For every plant that germinated, fresh tissue was collected, and genomic DNA isolated from ~20–30 mg lyophilized leaf tissue using a Maxwell Promega robot with the Maxwell RSC PureFood GMO and Authentication Kit (Promega, Madison, WI, USA).

PCR primer pairs were designed to bear at least two gene-specific SNP differences as compared to homologous genes. Primers used for PCR amplification and sequencing are detailed in Supplementary Table S1. We blasted all primers against the unmasked *Glycine max* ‘Williams 82’ genomic sequence (E-value cut off = 10.0) to ensure primer specificity (www.soybase.org, accessed on 15 November 2021). PCR amplification was performed using Titanium taq according to the manufacturer’s recommendations (Takara, Kusatsu, Japan) in a PTC-200 thermocycler (MJ Research/Bio-Rad, Hercules, CA, USA), using these conditions: 95 °C for an initial 5 min denaturation, followed by 40 cycles of 95 °C for 30 s, followed by 60 °C for 30 s, and an extension step at 72 °C for 1 min/kilobase of the target sequence. PCR products were separated on a 1% agarose gel and compared to DNA ladder size standards to ensure appropriate size. PCR products were purified through use of a QIAquick PCR purification kit (Qiagen, Hilden, Germany). After PCR purification, products were Sanger-sequenced at the DNA Core Facility of the University of Missouri-Columbia.

PCR product sequencing traces were imported into Geneious Primer software, version 11.0.11+9 (Invitrogen); trimmed; and aligned to gene sequences from the G275_W82.a2.v1 assembly (http://www.phytozome.net/soybean, accessed on 12 February 2022) using the Geneious Primer software, version 11.0.11+9 (Invitrogen). Disagreements were manually assessed in comparison to the G275_W82.a2.v1 assembly [39]. Putative indels and polymorphisms were verified by a minimum of two independent PCR reactions.

### 4.8. Immunoblot Analysis

Total seed proteins isolated from wild-type soybean cultivar ‘Maverick’ and T1:2 seeds of BBi-mutated CRISPR/CAS9 lines were resolved on 13.5% SDS-PAGE gels. Resolved proteins were transferred to nitrocellulose membranes and incubated with TBS (10 mM Tris-HCl, pH 7.5, 500 mM NaCl) containing 5% non-fat dry milk for 1 h at room temperature. Following this step, the nitrocellulose membranes were washed 3X with TBST (15 min each) and incubated overnight with 1:10,000 diluted antibodies raised either against purified soybean Kunitz trypsin or Bowman–Birk protease inhibitors. The 7S β-conglycinin and 11S glycinin-specific antibodies were used at 1:50,000 dilution. Specifically bound primary antibodies were detected using anti-rabbit IgG-horseradish peroxidase conjugates and a SuperSignal West Pico kit (Pierce, Rockford, IL, USA) as previously described [21].

### 4.9. Trypsin and Chymotrypsin Inhibitor Assay

Trypsin inhibitor activity in different BBi-mutated CRISPR/CAS9 lines was measured following the procedure described by [40]. Briefly, 20 mg of dry soybean seed powder was placed in a 2 mL Eppendorf centrifuge tube. To this, 1 mL of 10 mM NaOH solution was added and vigorously agitated on a vortex mixer for 10 min at room temperature. The resulting slurry was clarified by centrifugation at 16,000× *g* for 10 min. Clear supernatant was utilized for measuring KTi activity. Trypsin inhibitor activity was expressed as trypsin units inhibited (TUI) per mg of sample, and results are expressed as the mean ± SD from three biological replicates.

Chymotrypsin inhibitor activity in soybean seed extracts were measured N-Succinyl-Ala-Ala-Pro-Phe p-nitroanilide (AAPF) as the substrate. Soybean seed extract, which resulted in 40 to 60% chymotrypsin inhibition range, was added to a 1.5 mL Eppendorf tube containing 900 µL of assay buffer (100 mM-Tris-HCL, pH 8.0), 8 µL of α-chymotrypsin (Sigma-Aldrich Company) dissolved in 1 mM HCl solution (0.1 mg/mL), and seed extract (8 to 10 µL) and incubated at 37 °C for 5 min. Following this step, 80 µL of AAPF (1 mg/mL) was added and incubated for an additional 10 min at 37 °C. The assay was stopped by adding 500 µL of 30% acetic acid. Absorbance at 410 nm was measured, and chymotrypsin inhibitor units were calculated as the amount of inhibitor that reduced the absorbance at 410 nm by 0.1 optical density. The results are expressed as the mean ± SD from three biological replicates. Trypsin and chymotrypsin inhibitor assay data were visualized and compared using JMP statistical software 16.0 (SAS Institute Inc., Cary, NC, USA). One-way ANOVA was performed, and significant differences (α = 0.05) between means were determined using the Tukey–Kramer HSD test.

## Figures and Tables

**Table 1:** Analysis of trypsin inhibitor activity and chymotrypsin inhibitor activities in seeds from transgenic CRISPR-Cas9BBi-mutagenized and control plants. Tukey’s HSD indicates the results of a Tukey–Shapiro Honest Square Difference test; presence of the same letter indicates insignificant differences between genotypes.

		Seed Powder		Seed Protein	
Genotype	n=	Mean ± SD CIU/mg	Tukey’s HSD	Mean ± SD CIU/mg	Tukey’s HSD
‘Maverick’ (WT)	3	17.2 ± 1.91	A	56.93 ± 6.32	A
KTi-3	3	18.55 ± 0.42	A	60.97 ± 1.39	A
Event 5	3	15.93 ± 1.66	A	56.45 ± 5.89	A
Event 3	3	15.35 ± 1.63	A	51.34 ± 5.44	A
Event 4	3	6.2 ± 1.77	B	21.15 ± 6.03	B
Event 2	3	4.19 ± 1.03	B	15.28 ± 3.77	B
Event 7	3	3.9 ± 1.24	B	16.85 ± 5.38	B
Event 8	3	2.89 ± 0.04	B	11.54 ± 0.17	B
		**Seed Powder**		**Seed Protein**	
**Genotype**	**n=**	**Mean ± SD TIU/mg**	**Tukey’s HSD**	**Mean ± SD TIU/mg**	**Tukey’s HSD**
‘Maverick’ (WT)	3	57.25 ± 0.35	A	189.48 ± 1.17	A
KTi-3	3	36.45 ± 1.63	B	119.8 ± 5.35	BC
Event 5	3	35.7 ± 1.27	B	126.55 ± 4.51	B
Event 3	3	31.96 ± 1	C	106.88 ± 3.35	C
Event 4	3	24 ± 0.59	D	81.87 ± 2.01	D
Event 2	3	21.38 ± 0.06	DE	77.97 ± 0.21	D
Event 7	3	19.75 ± 1.89	E	85.35 ± 8.15	D
Event 8	3	14.46 ± 1.71	F	57.74 ± 6.82	E

## Data Availability

All data supporting the findings of this study are provided in the manuscript and Supplementary Information.

## Supplementary Materials

The supporting information can be downloaded at: https://www.mdpi.com/article/10.3390/ijms25115578/s1.
