# Supplementary material for: Bowman–Birk Inhibitor Mutants of Soybean Generated by CRISPR-Cas9 Reveal Drastic Reductions in Trypsin and Chymotrypsin Inhibitor Activities
Source: Int J Mol Sci. 2024 May 21;25(11):5578. doi: 10.3390/ijms25115578 (PMC11171862; doi:10.3390/ijms25115578)
Supplement: Supplementary file 1 [file ijms-25-05578-s001.zip › ijms-2986601-supplementary.pdf]

Bowman-Birk inhibitor mutants of soybean generated by CRISPR-Cas9 reveal drastic reductions in trypsin and chymotrypsin inhibitor activities

Won-Seok Kim [1], Jason D. Gillman [2], Sunhyung Kim[3], Junqi Liu [4], Madhusudhana R. Janga[5] , Robert M. Stupar[6], Hari B. Krishnan [7*]

Supplementary Materials

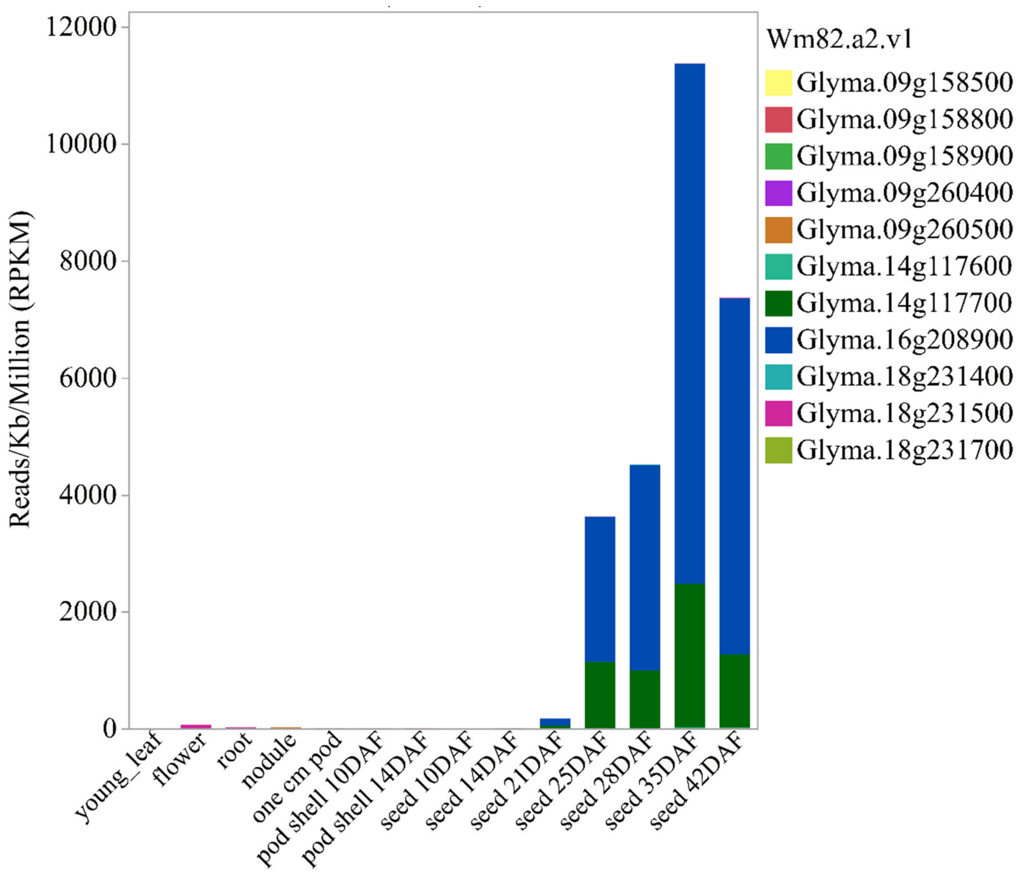

Figure S1. Gene expression of BBi genes in soybean. Data is repurposed from Severin et al. [20] using data housed on https://soybase.org/soyseq/. This RNAseq data was obtained from plant *P-C609-45-2-2*, a BC5F5 plant which was heterozygous for a seed protein QTL introgression from a *G. soja* (PI468916) into a *G. max* (A81-356022).

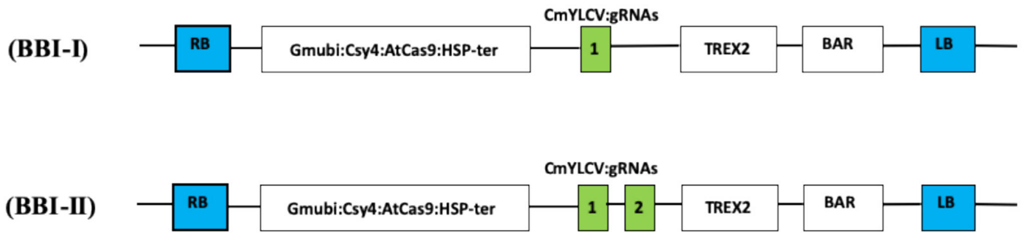

Figure S2. CRISPR-Cas9 cassettes targeting BBi genes. Events 5 was created using the BBi-II CRISPR-Cas9 construct and all other events were created through use of the BBi-I CRISPR-Cas9 construct.

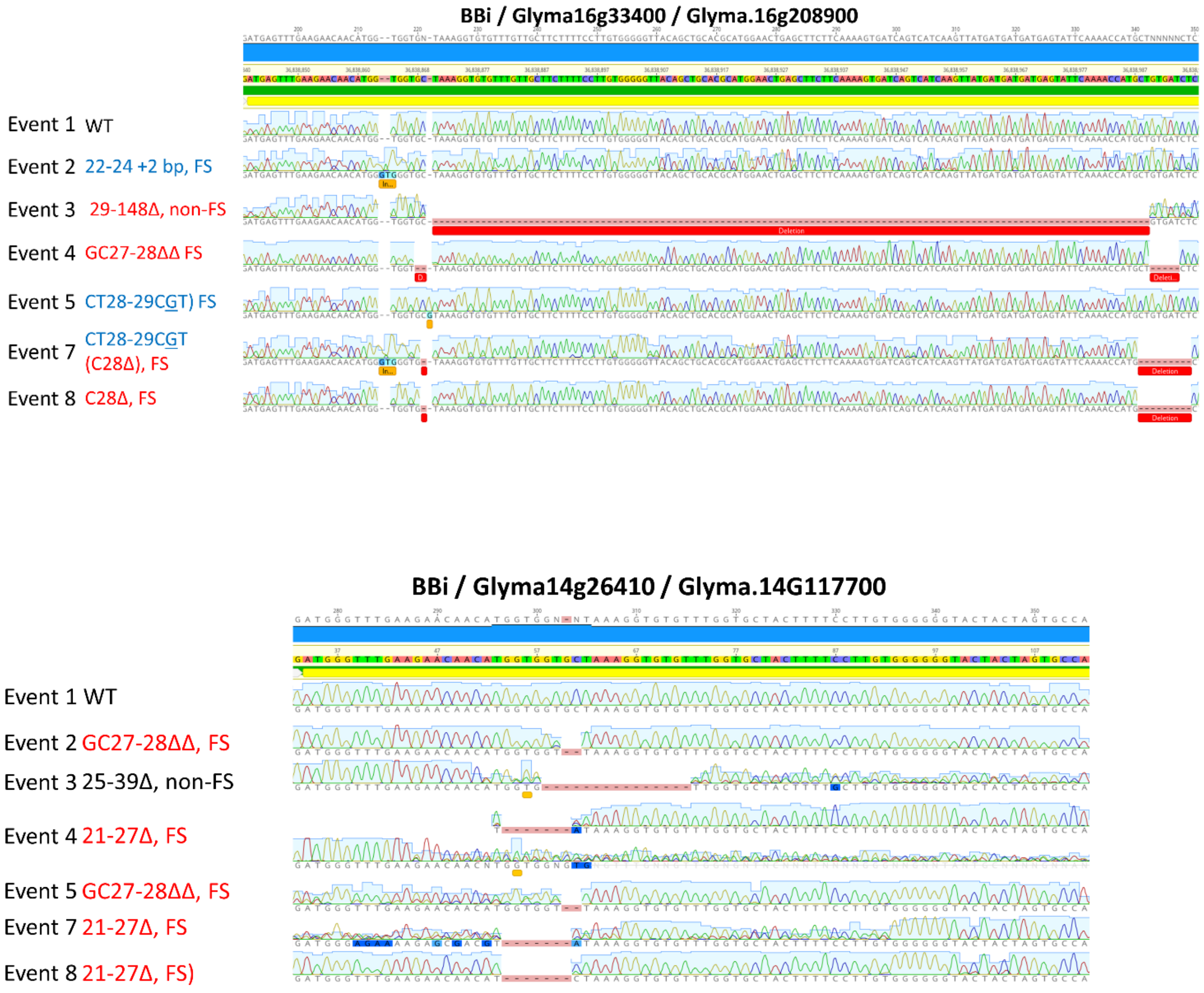

Figure S3. Sequencing traces for Glyma.16g208900 and Glyma.14G117700.

Table S1. Primers used for sequencing analysis and RT-PCR

Gene	Purpose	Primer name	Primer Sequence 5'- to -3'
Glyma.16g208900	PCR/sequencing	16G208900_WGF1	TAAATAACTCACTTCACGGGTTCT
Glyma.16g208900	PCR/sequencing	16G208900_WGR1	TGAACACACAAGTCGCATGA
Glyma.14G117700	PCR/sequencing	14G117700_WGF1	CAGTCATGATATCCTGCATGTGTG
Glyma.14G117700	PCR/sequencing	14G117700_WGR1	CCCATTGGAGAGAGAGCTTACAT
Glyma.16g208900	qRTPCR/sequencing	09G158500_WGF1	GAATTCGTGCCATTCAGCTTGCAAATC
Glyma.16g208900	qRTPCR/sequencing	09G158500_WGR1	AACAAAGCATAAAGGGGGCTTTGTCCG
Glyma.14G117700	qRTPCR/sequencing	14F117700_WGF1	GAATTCGTGCCATTCAGCTTGCAAATC
Glyma.16g208900	qRTPCR/sequencing	14F117700_WGR1	ACAAAGCCTAAAGGGGGCTTTGCCCA
Glyma.16g208900	qRTPCR/sequencing	09G158600_WGF1	AATTCATGTCACTCAGCTTGTGATCGC
Glyma.14G117700	qRTPCR/sequencing	09G158600_WGF1	CCTAAAGGGCTTTGTTCATTTGAGAGAG
Glyma.16g208900	qRTPCR/sequencing	09G158700_WGF1	AATTCATGTCACTCAGCTTGTGATCGC
Glyma.16g208900	qRTPCR/sequencing	09G158700_WGR1	CCTAAAGGGCTTTGTTCATTTGAGAGAA
Glyma.14G117700	qRTPCR/sequencing	16G208900_WGF1	AGCTTCTTCAAAAGTGATCAGTCATCAAGTTATG
Glyma.16g208900	qRTPCR/sequencing	16G208900_WGR1	GGCTGTGAGCGTGTGCACATACAGCTC
